# Das deutsche Klinikpartnerschaftsprogramm am Beispiel der Kooperation der Klinik für Augenheilkunde des Universitätsklinikums Düsseldorf mit der Fundación Visión in Asunción (Paraguay)

**DOI:** 10.1007/s00347-020-01183-6

**Published:** 2020-07-28

**Authors:** F. A. Steindor, M. Borrelli, E. Duarte, M. Roth, C. Holtmann, J. Menzel-Severing, R. Duerksen, G. Geerling

**Affiliations:** 1 Klinik für Augenheilkunde, Universitätsklinikum Düsseldorf, Heinrich-Heine Universität, Moorenstr. 5, 40225 Düsseldorf, Deutschland; 2 Fundación Visión, Ingavi Nº 8000 – Fernando de la Mora, Asunción, Paraguay

**Keywords:** Klinikpartnerschaft, Entwicklungshilfeprogramm, Deutschland, Paraguay, Hornhaut, Lamelläre Keratoplastik, DMEK, Hospital partnership, Development aid program, Germany, Paraguay, Cornea, Lamellar keratoplasty, DMEK

## Abstract

**Hintergrund:**

Seit 2014 besteht auf der Basis eines Memorandum of Understanding eine Partnerschaft zwischen der Universitätsaugenklinik Düsseldorf (UAK Düsseldorf) und der Fundación Visión (FV) in Asunción, die eine führende Rolle in der augenärztlichen Behandlung sowie der Prävention von Erblindung in Paraguay einnimmt. Seit 2016 existiert ein Programm zur Förderung internationaler Klinikpartnerschaften der Bundesregierung.

**Material und Methoden:**

Die Klinikpartnerschaft Düsseldorf–Asunción wird nach schriftlichem Antrag im Jahr 2016 durch das Ministerium für wirtschaftliche Zusammenarbeit gefördert. Im Rahmen der Projektlaufzeit sollen mithilfe mehrerer Aktivitäten in einem definierten Zeitplan moderne, minimal-invasive Hornhauttransplantationstechniken, die eine schnellere postoperative Rehabilitation und weniger Nachsorge erfordern, in Paraguay etabliert werden. Zusätzlich sollen erste Prävalenzdaten von Augenerkrankungen erhoben und die Basis für moderne Hornhautbanktechniken geschaffen werden.

**Ergebnisse:**

Zunächst erfolgte die Ausbildung eines paraguayischen Operateurs in der Durchführung von DMEK-Transplantationen in Deutschland. Zeitgleich wurden die instrumentellen Voraussetzungen für die Etablierung der DMEK-Technik in der FV geschaffen. Im September 2018 wurden unter Supervision eines erfahrenen OP-Teams aus Düsseldorf die ersten lamellären Hornhauttransplantationen durch das Team der FV erfolgreich in Paraguay durchgeführt. In der Folge wurden in Asunción auch die Verwendung einer Spenderhornhaut für je ein Transplantat für eine hintere und eine vordere lamelläre Keratoplastik (DMEK und DALK) umgesetzt. Im November 2019 erfolgte eine epidemiologische Datenerhebung zu Augenerkrankungen im Rahmen einer Feldkampagne der Fundación in einer ländlichen Region Paraguays. In deren Zug erlernte der datenerhebende Mitarbeiter der Universitätsaugenklinik Düsseldorf die Technik der manuellen Kleinschnittkataraktchirurgie. Im März 2019 wurde ein Mitarbeiter der FV in Düsseldorf in den Techniken der Hornhautgewebekultur und der Herstellung von Amnionmembrantransplantaten in der Lions-Hornhautbank NRW geschult.

**Schlussfolgerung:**

Mithilfe einer geförderten Klinikpartnerschaft können moderne minimal-invasive Hornhauttransplantationstechnik erfolgreich in die Augenklinik eines Schwellenlandes transferiert und im Gegenzug chirurgische Fertigkeiten für in Ausbildung befindliche Operateure aus Deutschland vermittelt werden. Damit stellt eine effiziente Klinikpartnerschaft die Grundlage für einen wechselseitigen Austausch dar und ist keine reine Einbahnstraße.

## Abstract (en)

**Background:**

A hospital partnership between the University Eye Hospital Düsseldorf (UAK Düsseldorf) and Fundación Visión in Asunción has existed since 2014 based on a memorandum of understanding. Fundación Visión plays a leading role in ophthalmological treatment and prevention of blindness in Paraguay. Since 2016 a program for the promotion of international hospital partnerships of the German government has been in place.

**Material and methods:**

Following a written application in 2016 the hospital partnership Düsseldorf-Asunción is funded by the Federal Ministry for Economic Cooperation in Germany. The aim of the project was to establish modern minimally invasive corneal transplantation techniques with faster postoperative rehabilitation and less follow-up in Paraguay with the help of several activities within a defined schedule. Secondary aims were to obtain first data on the prevalence of eye diseases in a typical patient cohort of the Fundación Visión and to establish an infrastructure for modern eye banking techniques in Paraguay.

**Results:**

First a Paraguayan surgeon was trained to carry out Descemet membrane endothelial keratoplasty (DMEK) transplantations in Germany. At the same time, the surgical instruments required for the DMEK technique were purchased and delivered to the Fundación Visión. In September 2018 the first lamellar corneal transplantations were successfully performed in Paraguay by the team from the Fundación Visión under the supervision of an experienced surgical team from Düsseldorf. Subsequently, the split cornea approach using a donor cornea for a posterior and an anterior lamellar keratoplasty (DMEK and DALK) was also implemented in Asunción. In November 2019 epidemiological data on eye diseases were collected during a field campaign of the Fundación Visión in a rural region of Paraguay. In the course of data collection an employee of the University Eye Hospital Düsseldorf learned small incision cataract surgery. In March 2019, a technician from the Fundación Visión was trained in Düsseldorf in corneal tissue culture techniques and preparation of amniotic membrane transplants in the Lions eye bank North-Rhine/Westfalia.

**Conclusion:**

With the aid of a supported hospital partnership modern minimally invasive corneal transplantation techniques can be successfully transferred to the eye hospital of a developing country and in return surgical skills can be taught to surgeons from Germany who are in training. Therefore, an efficient hospital partnership is the basis for a mutual exchange and is not purely one-way traffic.

Im September 2016 wurde die Initiative „Klinikpartnerschaften – Partner stärken Gesundheit“ vom Bundesministerium für wirtschaftliche Zusammenarbeit (BMZ) und der Else Kröner-Fresenius-Stiftung mit dem Ziel, humanitäre Partnerschaftsprojekte zwischen deutschen Organisationen des Gesundheitssektors und Partnern in Entwicklungs- und Schwellenländer zu vermitteln und zu fördern, gegründet. Die Deutsche Gesellschaft für Internationale Zusammenarbeit (GIZ) GmbH verantwortet im Zuge der Initiative Ausschreibungen und die Abwicklung von Fördermitteln und begleitet die Projekte hinsichtlich ihrer Umsetzung. Aktuell unterstützt die Initiative 182 Projekte mit 900 Partnern in 51 Ländern (Stand: 30.03.2020). Ein Teil dieser Projekte sind Klinikpartnerschaften zwischen deutschen Kliniken und größtenteils auf dem afrikanischen Kontinent gelegenen medizinischen Einrichtungen. Seit 2017 wird die Klinikpartnerschaft zwischen der Fundación Visión (FV) in Paraguay und der Universitätsaugenklinik Düsseldorf (UAKD) im Rahmen dieser Initiative durch das BMZ gefördert.

Paraguay liegt im Zentrum Südamerikas, hat eine mit Deutschland ungefähr vergleichbare Fläche, jedoch keinen direkten Zugang zum Meer. Es besteht für alle 7 Mio. Einwohner ein freier Zugang zu gesundheitlichen Versorgungsstrukturen. Aufgrund der geringen Bevölkerungsdichte des Landes und einer unausgewogenen Verteilung der Augenärzte mit Betonung auf der Hauptstadt hat ein großer Teil der Bevölkerung keinen Zugang zu einem Augenarzt (Tab. 1).

**Table 1:** 

	Deutschland	Paraguay
Landesgröße (km^2^)	357.582	406.752
Einwohnerzahl	83 Mio.	6,9 Mio.
Institution	Universitätsaugenklinik Düsseldorf	Fundación Visión Asunción inklusive Satelliten
Gesamtpatientenzahl (2019)	21.100	144.403
Gesamt-Operationszahl (2019)	4336	7562
Perforierende Keratoplastiken	83	50
Posteriore lamelläre Keratoplastiken (2019)	235	9

Die Fundación Visión (FV) wurde 1992 durch R. Duerksen gegründet und hat ihren Sitz in der Hauptstadt von Paraguay, Asunción. Sie ist eine gemeinnützige Stiftung und nimmt eine führende Rolle in der Behandlung und Prävention von Erblindung sowie der augenärztlichen Versorgung der Bevölkerung des Landes ein. Die FV verfügt über eine Zentralklinik, ebenfalls in Asunción, mit je einer Ambulanz für die allgemeine Bevölkerung und Privatpatienten, mit 11 Untersuchungseinheiten, einer Operationsabteilung mit 5 OP-Mikroskopen und einfachen Übernachtungsmöglichkeiten im Gebäude für aus entfernteren Regionen Paraguays anreisende Patienten. Die Ausstattung der Fundación entspricht weitgehend dem Standard einer deutschen Augenklinik. Perimeter, Ultraschall, Angiographie, Angio-OCT, Spiegelmikroskop, Laser u. a. sind vorhanden. Integriert in die FV sind eine Abteilung für Neuroophthalmologie, eine Low-vision-Abteilung, aber auch nichtaugenärztliche Angebote (z. B. Zahnheilkunde, HNO-Heilkunde u. a.). Im Jahr 2019 erfolgten in der Zentralklinik 74.388 konservative Patientenkontakte und ca. 4500 Operationen. Zusammen mit insgesamt 4 dauerhaft etablierten Satellitenkliniken und 20 bis 30 Feldkampagnen (z. B. in Schulen) in entlegeneren Landesteilen wurden insgesamt 144.000 Patienten betreut und ca. 7400 Operationen durchgeführt (Tab. 1).

Zusätzlich sind in der FV ein Ausbildungszentrum für jährlich 6 neue Weiterbildungsassistenten für 3 Jahre, eine Hornhautbank und als Spin-off 6 Apotheken und 9 Augenoptikergeschäfte etabliert. Die FV wird seit dem Jahr 2001 offiziell durch die Christoffel-Blindenmission jährlich finanziell unterstützt. Der größte Teil des Jahresbudgets wird jedoch durch Patientenbehandlungen erwirtschaftet. Allerdings zahlen lediglich ca. 30 % der Patienten den vollen Behandlungspreis; 50 % werden durch die Stiftung teilsubventioniert, und 20 % der Patienten werden völlig unentgeltlich behandelt.

Das operative Spektrum der FV reicht dabei von einfachen Kataraktoperationen über Glaukomoperationen bis hin zur Versorgung von Patienten mit Netzhautablösung mittels Vitrektomie. Hornhauttransplantationen zählten bislang schon zum OP-Spektrum der FV, wurden aber ausschließlich als perforierende Eingriffe durchgeführt. Für die Gewinnung von Spendergewebe betreibt die FV seit 2010 eine eigene Hornhautbank, die mit der Methode der Kurzzeitkultivierung arbeitet. Das so gelagerte Gewebe kann bis maximal zum 16. Tag post mortem verwendet werden. Trotz dieser guten strukturellen Gegebenheiten werden bislang nur wenige Transplantationen pro Jahr durchgeführt. Ursachen sind zum einen ein Mangel an Spendergewebe trotz der eigenen Hornhautbank, sodass regelmäßig Gewebe von nordamerikanischen Hornhautbanken bezogen werden muss, und zum anderen die für die Patienten z. T. große Distanz zum nächsten Augenarzt, sodass die postoperative Nachsorge erschwert ist.

Aufgrund des eigenen hornhautchirurgischen Schwerpunkts der Universitätsaugenklinik Düsseldorf wurde als Ziel der Kooperation daher die Etablierung neuer diagnostischer und therapeutischer Optionen für Hornhauterkrankungen gewählt.

## Material und Methoden

Initiiert wurde die Partnerschaft durch Vermittlung von V. Klauß (München), der den persönlichen Kontakt der ärztlichen Leiter beider Einrichtungen, R. Duerksen (Fundación Visión [FV]) und G. Geerling (UAKD) im Rahmen der Tagung, der Deutschen Ophthalmologischen Gesellschaft 2013 in Berlin herstellte. Im April 2014 erfolgte ein erster Vorortbesuch in Asunción, in dessen Rahmen sowohl ein Memorandum of Understanding unterzeichnet als auch die Zentralklinik und mehrere Satellitenkliniken der FV besucht wurden. Gefördert durch mehrere Kurzzeitdozenturen der DOG-Sektion für Internationale Ophthalmologie und den Verein der Freunde und Förderer der Universitätsaugenklinik Düsseldorf WiederSehen e. V., wurde zunächst im Jahr 2015 das Keimspektrum der Bindehautflora in einer Normal- und Glaukompopulation erhoben, eine Mitarbeiterin des Instituts für Mikrobiologie der Universidad Nacional de Asunción in der PCR-Technik als diagnostischem Pfeiler einer Akanthamöbenkeratitis in Düsseldorf ausgebildet und die Technik in Paraguay eingeführt [1].

Im Jahr 2017 wurde durch die Kooperationspartner gemeinsam ein Antrag auf Förderung der Klinikpartnerschaft an das BMZ gestellt und positiv beschieden. Bewilligt wurden insgesamt 48.000 € für Verbrauchsmaterial, Reisekosten und medizinische Geräte. Die Ziele der Anträge umfassten

1. die Etablierung lamellärer Hornhauttransplantationstechniken, um die Verwendung des limitiert vorhandenen Spendermaterials z. B. durch Teilung des Gewebes in ein anteriores (DALK) und posteriores (DMEK) lamelläres Hornhauttransplantat (Split-Technik) zu optimieren [2, 3],
2. die Vermittlung der Techniken der Gewebekultur, um die Versorgung mit qualitätskontrollierten Spendergeweben zu verbessern,
3. die Erhebung der Versorgungssituation einer Feldkampagne, um den Bedarf für zukünftige Kooperationsprojekte zu identifizieren.

**Figure 1:**
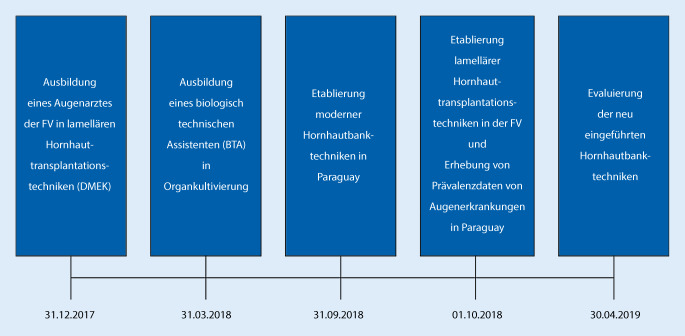

Alle Projektbestandteile wurden/werden durch die GIZ finanziert und observiert, jedoch eigenverantwortlich von den Projektpartnern umgesetzt. Die Aktivitäten werden nach einem definierten Zeitplan durchgeführt (Abb. 1).

## Ergebnisse

### Etablierung lamellärer Hornhauttransplantationstechniken

**Figure 2:**
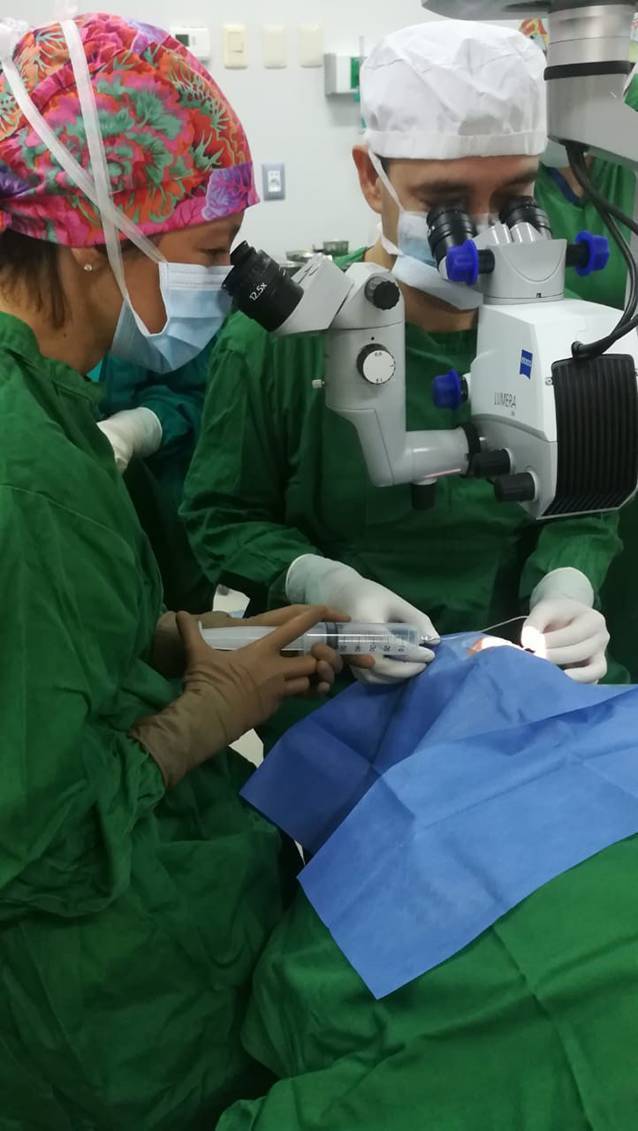

Der Beginn der Projektumsetzung verzögerte sich zunächst bedingt durch den Unfall eines Projektpartners und daraus resultierend ungeklärter Zuständigkeitsfragen. Im Februar 2018 erfolgte dann schließlich die Ausbildung eines erfahrenen Hornhautchirurgen aus Paraguay (ED) in der Präparation von DMEK-Transplantaten durch einen Besuch der UAKD. Das chirurgische Training erfolgte dabei an Hornhautspendermaterial, welches nicht für die Transplantation geeignet war und von der Lions-Hornhautbank NRW der UAKD zur Verfügung gestellt wurde. Darüber hinaus wurde das eigentliche Transplantationsverfahren durch Hospitation bei zahlreichen DMEK-Operationen während des zweiwöchigen Aufenthaltes demonstriert und die postoperative Nachsorge vermittelt. Zeitgleich wurden die erforderlichen OP-Instrumente aus Mitteln des Förderprogrammes in Asunción beschafft. Im September 2018 wurden schließlich unter Supervision durch ein in der DMEK-Technik erfahrenes OP-Team aus Ärztin und OP-Pflegekraft aus Düsseldorf die ersten drei DMEK-Operationen von dem so ausgebildeten Operateur erfolgreich in der Zentralklinik der FV durchgeführt (Abb. 2). Die postoperativen Verläufe gestalteten sich in allen Fällen regelrecht. Im Dezember 2018 wurde in der FV erstmals ein Hornhauttransplantat für 2 Patienten benutzt, in dem der in Düsseldorf ausgebildete Operateur zunächst die Descemet inklusive Endothel für eine DMEK benutzte und anschließend das verbliebende Stroma und Epithel für eine „deep anterior lamellar keratoplasty“ (DALK) verwendete.

### Vermittlung der Techniken der Gewebekultur

Ein BTA der FV war hierzu im Februar 2019 in Düsseldorf zu Gast. Seit Antragstellung hatte sich allerdings die Rekrutierung von Hornhautspendergewebe in Asunción verschlechtert, sodass eine erfolgreiche Etablierung der Organkultur fragwürdig erschien. Da die FV bislang nicht direkt über Amnionmembrantransplantate verfügte, wurde neben den Kulturtechniken für Spenderhornhäute daher auch die Präparation von Amnionmembrangeweben kurzfristig zusätzlich vermittelt. Dabei wurde gemeinsam mit den Mitarbeitern der Lions-Hornhautbank NRW eine komplette Charge von bis zu 150 Amnionmembrantransplantaten aus einer Spenderplazenta präpariert und die Herstellung des Lagerungsmediums vermittelt. Zurück in Asunción erstellte der Mitarbeiter des Projektpartners die erforderlichen SOPS. So kann die Amnionmembrantransplantation zukünftig als Alternative zur Hornhauttransplantation bei persistierenden Epitheldefekten oder Ulzera verwendet werden und so der Mangel von kornealem Spendergewebe umgangen werden.

Dennoch ist weiter geplant – wenn es der FV gelingt, durch Etablierung von Kooperationen mit neuen Spenderkliniken die Gewinnung von Hornhautspenden in Asunción wieder zu verbessern – in Zukunft die Organkultivierung einzuführen. Das Spendergewebe könnte dann bis zu 72 h post mortem entnommen und bei 34,7 °C bis zu maximal 6 Wochen in einem Brutschrank bis zur Transplantation kultiviert werden. Dieser – im Vergleich zur in der FV angewandten Kurzzeitkultivierung – deutlich längere Zeitraum würde es ermöglichen, den geeigneten Empfänger mit weniger Zeitdruck auszuwählen und vorzubereiten, während z. B. eine mikrobielle Kontamination des Spendergewebes mit höherer Sicherheit ausgeschlossen werden kann [4]. Zudem bleibt zu hoffen, dass durch die Weiterbildung des Personals der Hornhautbank der FV die Zahl von Entnahmen gesteigert und eine Unabhängigkeit von Importspendergewebe erreicht werden kann.

### Erhebung der Versorgungssituation einer Feldkampagne

**Figure 3:**
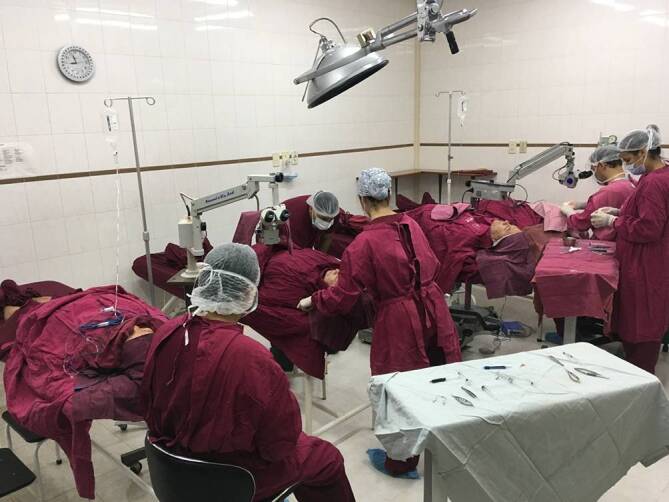

Im November 2018 besuchte ein Facharzt der UAKD die FV, um im Zuge einer sog. Campaña gemeinsam mit den paraguayischen Kollegen das Spektrum der in diesem Zusammenhang behandelten Augenerkrankungen zu erheben. Bei diesen mehrmals jährlich stattfindenden Feldkampagnen besucht ein Team der Fundación aus Operateuren und medizinischem Hilfspersonal die verschiedenen Provinzen Paraguays und untersucht im Rahmen einer offenen Sprechstunden die dort lebenden Menschen insbesondere auf das Vorliegen von Glaukom, Katarakt und diabetische Retinopathie. Die Untersuchung, konservative Maßnahmen und auch ggf. notwendige operative Eingriffe einer Campaña erfolgen entweder in einer der Satellitenkliniken der FV, Schulen oder einem Krankenhaus anderer Spezialisierung (z. B. Viszeralchirurgie, Unfallchirurgie oder Gynäkologie), wo dann vorübergehend ein augenärztlicher Operationssaal eingerichtet werden kann (Abb. 3).

**Figure 4:**
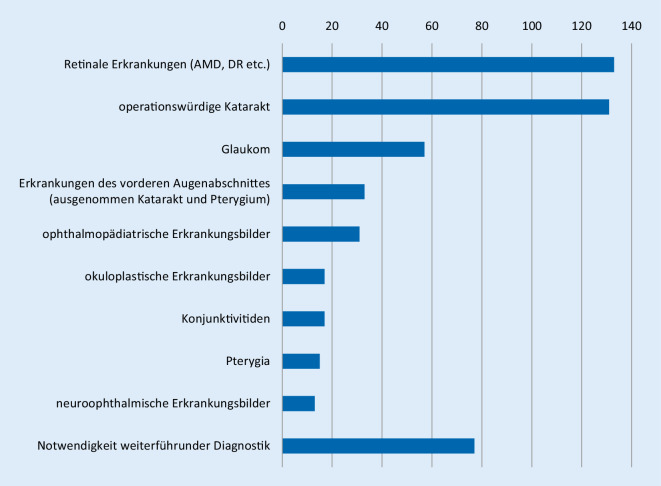

Während der begleiteten, fünftägigen Campaña wurden an den ersten 4 Tagen insgesamt 1185 Patienten untersucht. Davon waren 639 (53,9 %) Frauen und 546 (46,1 %) Männer (Abb. 3). Bei 133 (11,2 %) Patienten wurden retinale Erkrankungen (diabetische Retinopathie, altersbedingte Makuladegeneration etc.), bei 131 (11,1 %) eine operationswürdige Katarakt, bei 57 (4,8 %) ein Glaukom und in 15 Fällen (1,3 %) ein Pterygium diagnostiziert. Andere Erkrankungen des vorderen Augenabschnittes wurden in 33 (2,8 %), aus dem Bereich der Kinderophthalmologie in 31 (2,6 %), neuroophthalmologische Diagnosen in 13 (1,1 %) und der okulären Adnexe in 17 Fällen (1,4 %) festgestellt; 77 Patienten wurden zur weitergehenden Diagnostik mittels optischer Kohärenztomographie (OCT), 39-mal zum OCT der Makula und 38-mal der Papille, an die Zentralklinik in Asunción verwiesen (Abb. 4).

Bei den 131 Patienten mit operationswürdiger Katarakt wurde 115-mal (9,7 %) eine „small incision cataract surgery“ und 16-mal (1,4 %) eine Phakoemulsifikation geplant. Da trotz der Gemeinnützigkeit der FV die Patienten einen individuellen Anteil der Kosten selbst aufbringen müssen, wurden letztendlich nur 102 (8,6 %) Patienten auch in diesem Zeitraum operiert (46 Männer, 68 Frauen, Durchschnittsalter 67,4 ± 13,2; Abb. 2). Zusätzlich wurden 15 (1,3 %) Pterygiumexzisionen (7 Männer, 8 Frauen, Durchschnittalter 54,4 ± 12,7) durchgeführt (Abb. 3). Am letzten Tag der Campaña fanden lediglich postoperative Kontrollen sowie die Rückreise des Teams in die Hauptstadt Asunción statt. Im Rahmen der Campaña erlernte der Facharzt der UAKD die Technik der manuellen Kleinschnittkataraktchirurgie und führte erfolgreich 5 Eingriffe selber durch.

## Diskussion

Humanitäre Partnerschaftsprojekte in Entwicklungs- und Schwellenländer werden unterschiedlich gefördert und gelebt. Das Programm des BMZ für Klinikpartnerschaften, hier am Beispiel der Kooperation zwischen der Fundación Visión/Paraguay und der Universitätsaugenklinik Düsseldorf dargestellt, zeichnet sich neben der konkreten Zielgruppe der Kliniken durch vertraglich festgehaltene Aktivitätszeitpunkte und Förderzeiträume aus. Die FV selber wiederum ist durch eine europäisch geprägte Organisationsstruktur und exzellente apparative Ausstattung charakterisiert. Der Gründer der FV ist Teil einer größeren deutschstämmigen und auch in der 4. Generation noch deutschsprachigen Minderheit in Paraguay. Dies wie auch die sonst spanische Landessprache erleichtert entsprechend sprachmächtigen Teilnehmern die direkte Kommunikation mit dem Projektpartner und Patienten. Die FV verfügt nicht nur über das Instrumentarium für die Phakoemulsifikation und Vitrektomie, sondern auch über eine eigene Hornhautbank, einen Excimerlaser und einen Simulator für das Training von Assistenzärzten mittels virtueller Kataraktoperationen. Ihre Ausstattung ist damit deutlich besser als in vielen z. B. afrikanischen Kliniken. Durch diese in der Zentralklinik in Asunción etablierte gute Versorgungsstruktur und die Ausbildung von Assistenzärzten aus zahlreichen anderen Ländern Südamerikas ist daher über die unmittelbare Ausbildung einzelner Personen in Deutschland hinaus ein Multiplikationseffekt und damit eine Verbesserung der Versorgung für eine größere Zahl Patienten zu erwarten. Paraguay ist darüber hinaus politisch seit Jahrzehnten eine stabile Demokratie. Trotz dieser Voraussetzungen ergaben sich im Zuge des Klinikpartnerschaftsprojektes Abweichungen und Verzögerungen in der Umsetzung, die jedoch eher durch persönliche Lebensumstände als durch strukturelle und Kommunikationsprobleme ausgelöst waren.

Trotz der sehr guten Ergebnisse sind lamelläre endotheliale Keratoplastiken, insbesondere DMEK, in sich entwickelnden Ländern eine bisher kaum angewandte Technik [5, 6]. Da im Vergleich zur DSAEK für die DMEK kein Mikrokeratom oder andere teure technische Ausstattung angeschafft werden muss, ist die lange Lernkurve in Verbindung mit teilweise nur wenigen pro Jahr verfügbaren Transplantaten (z. B. ca. 64 Transplantationen im Jahr 2013 in Indonesien bei ca. 250 Mio. Einwohnern) vermutlich das größte Hindernis einer weiteren Verbreitung der Technik [7, 8]. Wenn aber bereits erfahrene Operateure – wie im Rahmen der hier vorgestellten Kooperation erfolgt – lediglich gezielt in einer speziellen Technik geschult werden müssen, kann die Lernkurve möglicherweise verkürzt und Barrieren, die Technik in der Heimat zu verwenden, können abgebaut werden. Die Vorteile der minimal-invasiven DMEK-Technik könnten insbesondere in sich entwickelnden Ländern zu einer Verbesserung der Versorgungssituation beitragen, denn der Nachsorgeaufwand (z. B. Fadenzug) und auch das Risiko postoperativer Infektionen oder Abstoßungsreaktionen ist hier geringer. Darüber hinaus können bei Spendermangel – wie in diesem Fall nur 1 Monat nach dem Besuch in Asunción erfolgreich umgesetzt – aus einer Spenderhornhaut mehrere Transplantate zur Versorgung von 2 Empfängern im Rahmen einer anterioren (DALK) und einer posterioren (DMEK) lamellären Hornhauttransplantation hergestellt werden (Split-Technik). So kann die Etablierung der DMEK-Technik einen Multiplikatoreffekt beim Abbau von Wartelisten auch in einem Entwicklungs- oder Schwellenland haben [2, 3].

Zusätzlich muss betont werden, dass die hier vorgestellte Kooperation keine „Einbahnstraße“, sondern einen bidirektionalen Wissenstransfer darstellt, da neben dem Ausbau der Versorgungsstrukturen der FV auch ein Mitarbeiter der UAKD die Technik der manuellen Kleinschnittkataraktchirurgie im Rahmen eines Besuchs in Paraguay erlernen konnte.

Mithilfe einer geförderten Klinikpartnerschaft können moderne minimal-invasive Hornhauttransplantationstechniken wie die DMEK erfolgreich in der Augenklinik eines Schwellenlandes eingeführt werden. Der Ausbau bestehender lokaler Strukturen hat v. a. im Sinne der Nachhaltigkeit entscheidende Vorteile im Vergleich zu Kurzzeiteinsätzen ausländischer Operateure. Jedoch bleibt auch bei langfristigen Klinikpartnerschaften der Erfolg von lokalen ökonomischen, kulturellen, politischen Faktoren und letztendlich vom Engagement der handelnden Personen abhängig.
